# Identification of Candidate Therapeutic Genes for More Precise Treatment of Esophageal Squamous Cell Carcinoma and Adenocarcinoma

**DOI:** 10.3389/fgene.2022.844542

**Published:** 2022-05-19

**Authors:** Aneta Polewko-Klim, Sibo Zhu, Weicheng Wu, Yijing Xie, Ning Cai, Kexun Zhang, Zhen Zhu, Tao Qing, Ziyu Yuan, Kelin Xu, Tiejun Zhang, Ming Lu, Weimin Ye, Xingdong Chen, Chen Suo, Witold R. Rudnicki

**Affiliations:** ^1^ Institute of Computer Science, University in Bialystok, Białystok, Poland; ^2^ Department of Epidemiology, School of Public Health, Fudan University, Shanghai, China; ^3^ Fudan-Taizhou Institute of Health Sciences, Taizhou, China; ^4^ State Key Laboratory of Genetic Engineering and Collaborative Innovation Center for Genetics and Development, School of Life Sciences, Fudan University, Shanghai, China; ^5^ Clinical Epidemiology Unit, Qilu Hospital of Shandong University, Jinan, China; ^6^ Department of Medical Epidemiology and Biostatistics, Karolinska Institute, Stockholm, Sweden; ^7^ Shanghai Institute of Infectious Disease and Biosecurity, Shanghai, China; ^8^ Computational Centre, University of Bialystok, Białystok, Poland

**Keywords:** esophageal cancer (ESCA), drug target genes, Feature Selection (FS), random forest (RF), ensemble learning (EL)

## Abstract

The standard therapy administered to patients with advanced esophageal cancer remains uniform, despite its two main histological subtypes, namely esophageal squamous cell carcinoma (SCC) and esophageal adenocarcinoma (AC), are being increasingly considered to be different. The identification of potential drug target genes between SCC and AC is crucial for more effective treatment of these diseases, given the high toxicity of chemotherapy and resistance to administered medications. Herein we attempted to identify and rank differentially expressed genes (DEGs) in SCC *vs*. AC using ensemble feature selection methods. RNA-seq data from The Cancer Genome Atlas and the Fudan-Taizhou Institute of Health Sciences (China). Six feature filters algorithms were used to identify DEGs. We built robust predictive models for histological subtypes with the random forest (RF) classification algorithm. Pathway analysis also be performed to investigate the functional role of genes. 294 informative DEGs (87 of them are newly discovered) have been identified. The areas under receiver operator curve (AUC) were higher than 99.5% for all feature selection (FS) methods. Nine genes (i.e., ERBB3, ATP7B, ABCC3, GALNT14, CLDN18, GUCY2C, FGFR4, KCNQ5, and CACNA1B) may play a key role in the development of more directed anticancer therapy for SCC and AC patients. The first four of them are drug targets for chemotherapy and immunotherapy of esophageal cancer and involved in pharmacokinetics and pharmacodynamics pathways. Research identified novel DEGs in SCC and AC, and detected four potential drug targeted genes (ERBB3, ATP7B, ABCC3, and GALNT14) and five drug-related genes.

## Introduction

Esophageal cancer (SCA) is a very aggressive condition. In 2018, there were an estimated 17,290 new cases of esophageal cancer and 15,850 deaths in the United States alone (Noone et al., 2018). Although its prognosis has gradually improved due to advances in treatment and surgical techniques, the overall survival remains poor, with only 10–22% patients showing survival of >5 years after diagnosis in Europe, the United States, and China (Dubecz et al., 2012). Such a low survival outcome is mainly attributable to late diagnosis and lack of effective treatment methods.

Esophageal cancer represents a heterogeneous group of cancers and consists of two main histological subtypes: squamous cell carcinoma and esophageal adenocarcinoma. Generally, SCC is associated with worse prognosis than AC (Enzinger and Mayer, 2003), but it is dependent on cancer progression (Shimada et al., 2013). SCC and AC are increasingly being considered as separate conditions with different etiologies, epidemiology, histopathology, and other biological behavior (Tustumi et al., 2016; Lagergren et al., 2017). Furthermore, recent studies have reported distinct differences in their genomic profiles (Wang et al., 2015; Salem et al., 2016; Network, 2017), and the number of different biomarkers between SCC and AC is in the order of thousands (Greenawalt et al., 2007; Lin et al., 2017). Analyses involving The Cancer Genome Atlas (TCGA) Research Network have shown that with respect to the overall genomic landscape, SCC and AC are more similar to non-esophageal cancers than to each other (Network, 2017; Salem et al., 2018).

Despite profound biological and clinical differences between SCC and AC, the standard therapy and drugs used in chemotherapy remain largely similar (Lordick et al., 2016). A combination of platinums, taxanes, anthracyclines, or pyrimidine analogs is usually prescribed to patients with esophageal cancer, regardless of the pathological subtypes (Abdo et al., 2017; Davidson et al., 2017). Bang et al. (2010) reported that therapies targeting *HER2* (trastuzumab) and vascular endothelial growth factor receptor 2 (ramucirumab) are highly effective for gastroesophageal junction cancer. Davidson et al. (Davidson et al., 2017)found that patients with AC showed a significantly higher response rate to first-line fluoropyrimidine-based chemotherapy than those with SCC. Earlier identification of drug-related genes with a high difference in their expression levels between SCC and AC can be helpful for understanding the differences in the clinical response of patients with esophageal cancer to different anticancer drugs, given the high toxicity of chemotherapy and resistance to administered medications. For instance, (Abdo et al., 2017) suggested that information pertaining to the overexpression of genes encoding drug molecular targets could help oncologists in decision making; the screening of nine genes (*HER2, EGFR, PD-L1, ERCC1, TUBB3, TS, RCF, TOPOI,* and *TOPO2A*) was recommended to ensure more effective immunotherapy and chemotherapy outcomes in patients with SCA.

Herein we aimed to identify novel biomarkers with the intention of improving diagnosis, as well as potential drug target genes and molecular candidate drugs to achieve effective treatment of SCC and AC. We used the heterogeneous ensemble feature selection method to identify the most informative biomarkers for the classification of the subtypes of esophageal cancer and the random forest machine learning algorithm (Breiman, 2001) to evaluate the quality of the set of the selected features. The ensemble filter method is based on six diverse filtering FS methods for reducing the risk of omitting biological relevant biomarkers. Such advanced machine learning methods have not been previously used for the classification of SCC and AC. Furthermore, we primarily focused on specific targets of drug action, such as membrane proteins, which are pivotal for drug development, because most therapeutics target membrane proteins are responsible for altering cellular signaling. We specifically studied membrane proteins affected by differentially expressed genes (DEGs) between SCC and AC and characterized relevant genes, which should enable individualized drug development. Additionally, we analyzed gene-gene interactions using the GeneMANIA software (Warde-Farley et al., 2010).

## Materials and Methods

### Preprocessing and Integration of Datasets

Gene-level RNA-seq analyses of esophageal carcinoma were performed (two experiments): RNA-sequencing data from TCGA esophageal cancer project (TCGA-SCA, https://portal.gdc.cancer.gov/projects/TCGA-ESCA) and NODE-SCC data deposited in the National Omics Data Encyclopedia of China (accession no.: OEP000138, http://www.biosino.org/node/project/detail/OEP000138). TCGA-SCA mRNA data have been previously investigated (Zeng et al., 2017), but the main analysis was focused on the identification of molecular targets for prognostic analysis and diagnosis with reference to normal esophageal tissues (Zhan et al., 2016), not for the classification of SCA subtypes. TCGA-SCA dataset contains data of White and Asian patients; patients with SCC (*n* = 87, 87% male) had a median age of 57 years (range, 36–90) and those with AC (*n* = 71, 83% male) had a median age of 71 years (range, 27–86). The second dataset was generated by the Fudan University, containing data of 43 tumor tissue samples obtained from Asian patients with SCC (75% male) with a median age of 69 years (range, 50–82).

Data pre-processing and all analyses were conducted using the open-source statistical software R v3.4.3 (R Core Team, 2017a). Data preparation includes four main subprocesses: cleaning, transformation, merging, and reduction. TCGA-SCA dataset contains gene level RNA-seq data of 158 tumor tissue samples and 20,501 biomarkers, whereas the NODE-SCC dataset contains data of 43 tumor tissue samples obtained from patients with SCC and 21,309 biomarkers.

After cleaning up and Log2 transformation of the data, both datasets were subjected to unsupervised biomarker set selection. For this purpose, the following criteria were applied using the R package genefilter (Gentleman et al., 2020): (1) robust coefficient of variation of RNA-seq expression level (GE) > 0.05 and (2) at least 10% samples having GE > 0.45 (the number of biomarkers rapidly decreases below this GE threshold) for TCGA-SCA dataset and −4.35 for the NODE-SCC dataset. The software package BRB-ArrayTools (Simon, 2020) includes a detailed description of this reduction procedure. These standard preprocessing procedures are particularly important when using statistical methods because RNA-tags with low expression measurement range are not normally distributed. TCGA-SCA and NODE-SCC datasets were merged (COM-SCA dataset); i.e., the pairs of biomarkers belonging to the same gene were integrated. The COM-SCA dataset contains 201 samples (130 patients with SCC and 71 with AC) and 16,596 overlapping biomarkers. The ComBat function in “SVA” R package (Leek et al., 2018) was used for removing batch effects between the two experiments and races.

### Statistical Analysis

To quantify feature distribution in COM-SCA dataset, the statistical analysis was performed. The 67% DEGs in SCC group and 61% DEGs in AC group have a fairly symmetrical distribution of data and the value of skewness ranges from -0.5 to 0.5. The Levene’s test showed that 74% DEGs have variances equal in these groups, the Bartlett test showed 60%. The Kolmogorov-Smirnov test confirmed the normality distribution in 86% DEGs in both groups of patients. Considering the above, the normal distribution of biomarkers in both groups was assumed. The Welch *t*-test was used for the differential expression analysis of RNA-seq data, as one of six used feature selection methods (Supplementary Table S1).

### Feature Selection and Prediction Model Building

To validate the FS process, machine learning models for discerning SCC from AC were built using selected markers as explanatory variables. To this end, we applied the random forest algorithm (Breiman, 2001) as implemented in the randomForest package (Liaw and Weiner 2002) in R (R Core Team, 2017b). Random forest is considered to be one of the best off-the-shelf classifier algorithms that can be applied to nearly all classes of problems. The conclusion of a very thorough study devoted to testing multiple algorithms on numerous publicly available datasets (Fernandez-Delgado and Cernadas., 2014) was that random forest is the best overall classification algorithm, that generally gives good results, very rarely gives bad results, and in many cases gives best ones. These conclusions were based on analysis of results of the application of 179 algorithms belonging to 17 broad families of algorithms on 121 diverse datasets.

Considering the problem of an unbalanced dataset (Luque et al., 2019), the area under the receiver operator characteristic curve and Matthews correlation coefficient (MCC) were used as measures of classification performance.

**FIGURE 1:**
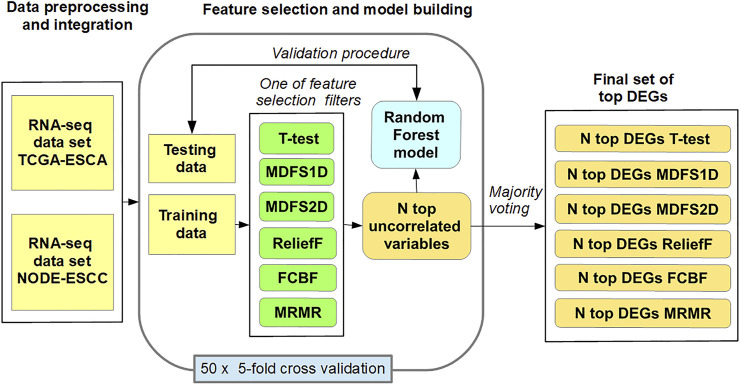
Procedures involved in selecting the most informative biomarkers.

Figure 1 shows the methods used for the identification of the most informative biomarkers and building prediction RF models. Individual RF models from RNA-seq data were constructed in 50 loops with the following procedure:

(1) The dataset was randomly split into five equal partitions;
(2) Insignificant genes between SCC and AC were ranked/filtered out using Ttest, MDFS1D, MDFS2D, FCBF, MRMR, and ReliefF on four partitions (training set);
(3) Highly correlated features (Spearman’s rank correlation coefficient >0.7) were rejected from the ranked list;
(4) Random forest classifier was built on the training set using the top-N features;
(5) Model quality was evaluated on the remaining partition (test set);
(6) Steps 2–5 were repeated for all k-partitions and each FS method.

The final number of top features used for model building was experimentally established. In addition, the quality of predictive RF models and stability of feature selection as a function of the number of top features were measured for all FS methods. The stability of feature selection was gauged by the similarity of different sets of relevant variables in cross-validations with the Lustgarten stability measure (ASM) (Lustgarten et al., 2009). All FS methods used the same cross-validation splits.

### Identification of Key Genes

The complete list of key genes was derived using the following procedure:

(1) Top-N DEGs were selected from each of the 250 ranked lists for each FS method independently;
(2) A set of N genes with the highest frequency of occurrence among the 250 lists was identified for each FS method independently;
(3) From the six gene sets corresponding to the FS methods, key genes were selected;
(4) Log2 fold change (FC) of normalized RNA-seq gene expression between SCC and AC was calculated using the formula Log2FC = Log2 (GE_SCC_/GE_AC_), wherein GE_SCC_ and GE_AC_ represent the mean value of normalized RNA-seq gene expression level for SCC and AC, respectively, for each gene. The key gene list was then sorted according to absolute Log2FC values;
(5) Membrane protein-encoding genes and their association with well-known drugs were subsequently identified.

The Human Protein Atlas database (Uhlen et al., 2015) was used for selecting membrane protein-encoding genes. Data pertaining to drugs and drug–gene interactions were collected from several databases, namely, DGIdb (Cotto et al., 2018), DrugBank (Wishart et al., 2006), and Therapeutic Target DB (Wang et al., 2020), and additional information was obtained from ApexBio, FDA Approved Drugs, ClinicalTrials.gov, PharmGKB, and GeneCards.

## Results

### Informative Biomarkers

We investigated the molecular markers that could distinguish between the two main histological subtypes of esophageal cancer. To identify DEGs from the full combined RNA-seq datasets (COM-SCA), we used six feature filters, namely Welch *t*-test (Ttest) (Welch, 1947), one- and two-dimensional FS filters based on information theory (MDFS1D and MDFS2D, respectively) (Piliszek et al., 2019), fast correlation-based filter (FCBF) (Yu and Liu, 2003), the ReliefF algorithm (Kononenko, 1994), and minimum redundancy and maximum relevance (MRMR) (Ding and Peng, 2005).

Using the methods Ttest, MDFS1D, and MDFS2D, which could identify relevant predictor features but did not remove redundant ones, 7142 unique relevant genes were identified (refer to the Venn diagram in Supplementary Figure S1) in the entire data set.

The other three FS methods, namely ReliefF, MRMR, and FCBF, either returned just a ranking of features (ReliefF, MRMR) or a set of top non-redundant informative ones (MRMR and FCBF). In particular, FCBF identified only 59 relevant variables, all of which were found by all other algorithms as well.

MDFS1D identified the highest number of relevant features (5437), and this number was used as the limit of relevant variables returned by MRMR and ReliefF. The final number of unique DEGs identified by at least one method was 8246. Although this number is bound to include false positive data, it shows how distinct SCC and AC are at the molecular level.

A much smaller number of features is sufficient to build a machine learning model that can distinguish between SCC and AC with high precision levels (Supplementary Figures S2, S3). In the current study, a high average predictive power of random forest model (AUC = 0.994) was already achieved for 20 features for all filters. However, small sets of most relevant features showed instability in 5-fold cross-validation repeated 50 times (Supplementary Figure S4). For all algorithms, except FCBF, the maximal stability value of the Lustgarten measure for sets of top N features as N approaching 100 (Table 1). In contrast, the FCBF method attempts to minimize redundancy in the set of features. This optimization increases instability, because it amplifies small random differences in relevance observed in the different repeats of cross-validation. ASM values, indicating stability, were <0.6 in the entire studied range of top-N uncorrelated features. These instabilities could be attributed to a high number of highly relevant variables with very similar levels of relevance. Random fluctuations due to differences in the composition of samples in cross-validation lead to large changes in the relative rankings of features in different samples. To minimize the influence of these fluctuations, top 100 features from each algorithm were used for further analysis, ensuring that most relevant genes were a part of them. The good predictive power for all methods (Table 1) and the small overlap between the six sets of top 100 genes from the six algorithms suggested that each algorithm identified different aspects of relevance.

**TABLE 1:** Comparison of feature selection methods.

Metric	Ttest	MDFS1D	MDFS2D	FCBF	ReliefF	MRMR
AUC	0.996	0.998	0.997	0.994	0.996	0.999
MCC	0.994	0.997	0.996	0.991	0.993	0.998
ASM	0.52	0.43	0.40	0.05	0.34	0.53

Note: The first two rows display AUC and MCC obtained for RF classifier on 100 most relevant genes selected with each feature selection method. The last row displays the stability of these, which was measured using ASM. Fifty repeats of 5-fold cross-validation were performed. Standard deviation of mean AUC and MCC was <0.001. See notation in the main text.

The final list of relevant genes ultimately included six lists of 100 genes identified by six independent FS methods. Overall, 294 genes represented the key set of biomarkers that could be used to distinguish between AC and SCC. The complete list of genes is shown in Supplementary Table S1. More than 46% genes in this set showed a high difference in expression levels between AC and SCC, with abs (Log2FC) ≥ 3.0 (FC = fold change between SCC and AC). Under-expressed genes were the most prevalent (59%).

### Potential Therapeutic Targets

Herein we focused on membrane protein-encoding genes that are one of the most important macromolecules for drug development. In total, 84 genes from the list of the most important biomarkers were labeled as “membrane proteins” or “predicted membrane proteins” in the Human Protein Atlas database. Of these 84 genes, 44 were related to drugs (Supplementary Table S2).

**FIGURE 2:**
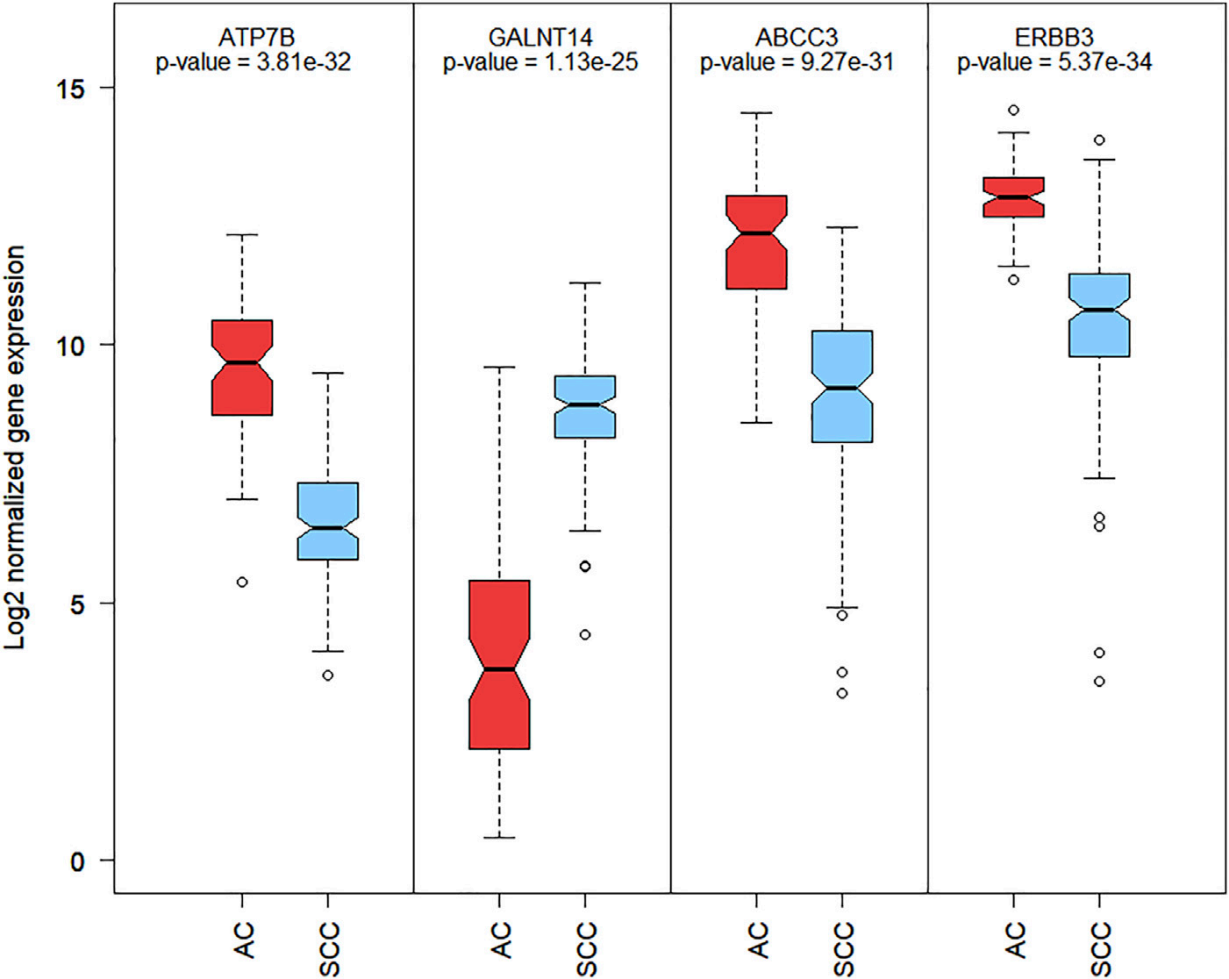
Boxplot of Log2 normalized RNA-Seq gene expression of 4 membrane encoding genes related with SCA anti-cancer drugs. Boxplot contains the *p*-value of mean differential expression between AC and SCC patients groups with a two-sample *t*-test.

**FIGURE 3:**
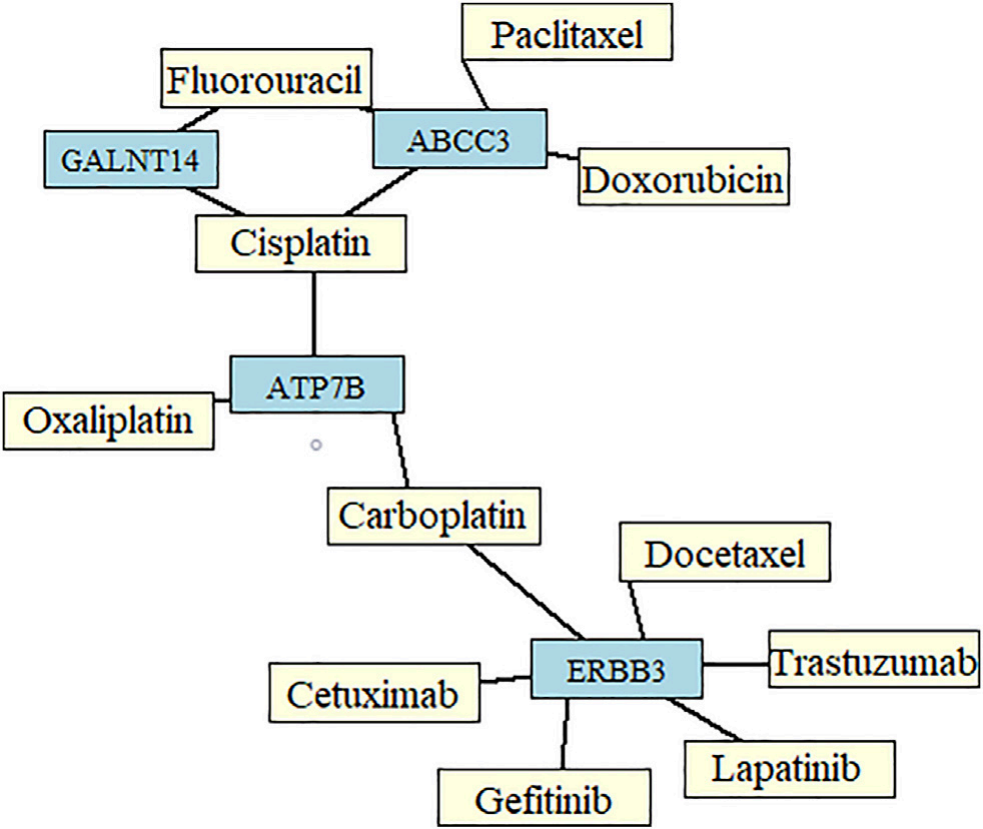
Network of drugs important for chemotherapy in patients with esophageal cancer, and genes identified from the set of the most informative biomarkers predictive of the two main histological subtypes of esophageal cancer.

The most common drugs used for anticancer therapy in the case of patients with SCA include carboplatin, paclitaxel, platinol, epirubicin, docetaxel, fluorouracil, oxaliplatin, irinotecan, cetuximab, lapatinib, trastuzumab, doxorubicin, cisplatin, leucovorin, capecitabine, gefitinib, ramucirumab, mitomycin, bleomycin, and amethopterin (Abdo et al., 2017; Huang and Yu, 2018). We found that four of the 84 membrane protein-encoding genes were drug targets for chemotherapy and immunotherapy of esophageal cancer and involved in pharmacokinetics and pharmacodynamics pathways. Three genes were under-expressed Erb-B2 receptor tyrosine kinase 3 (*ERBB3*), ATPase copper-transporting beta (*ATP7B*), and ATP-binding cassette subfamily c member 3 (*ABCC3*) and one was overexpressed polypeptide N-acetylgalactosaminyltransferase 14 (*GALNT14*) in SCC *vs*. AC. Among these four genes, *GALNT14* showed the highest difference in expression levels between SCC and AC (Log2FC = 4.62, Figure 2). A network of anticancer drugs related to the four genes is shown in Figure 3.

To identify new potential therapeutic targets that may affect the choice of SCA therapy, we identified genes with the highest difference in expression levels between SCC and AC, and arranged them according to the absolute value of Log2FC for the 84 genes predicted to encode membrane proteins. Biological functions of each of these genes are listed in Supplementary Table S2. Top 10 over/under-expressed genes in SCC *vs*. AC and examples of drugs associated with them are shown in Table 2. Although these drugs are not necessarily chemotherapy drugs, they provide new insights into targeted therapy for SCC and AC.

**TABLE 2:** Top 10 membrane protein-encoding genes that were under- or overexpressed in SCC *vs*. AC

Top down-expressed membrane protein encoding genes in SCC *vs*. AC			
No	Gene	Log2FC	Drugs
1	*CLDN18*	7.57	CLAUDIXIMAB
2	*TM4SF5*	7.27	
3	*SLC6A20*	6.03	
4	*TM4SF4*	5.87	
5	*SI*	5.86	ACARBOSE, SCOPOLAMINE, DEXAMETHASONE, MIFEPRISTONE, STREPTOZOTOCIN, FURAN, SODIUM BETA-NICOTINAMIDE ADENINE DINUCLEOTIDE PHOSPHATE, HEXAMETHYLENEBISACETAMIDE
6	*GPR128*	5.77	
7	*GUCY2C*	5.49	LINACLOTIDE, PLECANATIDE, PANITUMUMAB, PIRIBEDIL (CHEMBL1371770), PHOSPHORIC ACID, LINACLOTIDE ACETATE, GUANOSINE MONOPHOSPHATE, CYCLIC GMP
8	*CLDN2*	5.29	CALCIUM
9	*GPR35*	5.16	PROSCILLARIDIN, BUMETANIDE, FUROSEMIDE, TRANSTORINE, PAMOIC ACID, ZAPRINAST, PYRANTEL, KYNURENIC ACID
10	*MIA2*	5.06	

Top up-expressed membrane protein encoding genes in SCC *vs*. AC			
No	Gene	Log2FC	Drugs
1	*DSC1*	4.64	CALCIUM
2	*GALNT14*	4.62	FLUOROURACIL, CARBOQUONE, MITOXANTRONE, CISPLATIN, CALCIUM, MANGANESE
3	*DMRT2*	4.61	
4	*SLC35F3*	4.26	
5	*KCNQ5*	4.24	CELECOXIB, IRINOTECAN, EZOGABINE, TEDISAMIL, FLINDOKALNER, CHEMBL317935, LINOPIRDINE, CHEMBL342375, DALFAMPRIDINE, GUANIDINE HYDROCHLORIDE, NERISPIRDINE, POTASSIUM, TETRAETHYLAMMONIUM
6	*RYR1*	3.91	DANTROLENE SODIUM, SURAMIN, MAGNESIUM, cA2, ADENOSINE TRIPHOSPHATE, CAFFEINE, PROCAINE, RYANODINE, DANTROLENE, SURAMIN, RUTHENIUM RED, CAFFEINE, DANTROLENE, TETRACAINE
7	*SLC6A2 (SLC6A5)*	3.75	AMPHETAMINE, GUANADREL, GUANETHIDINE, REBOXETINE, MIRTAZAPINE, LOXAPINE, DOXEPIN, AMOXAPINE, MAZINDOL, ERGOTAMINE, COCAINE, PHENMETRAZINE, SIBUTRAMINE, NOMIFENSINE, CHLORPHENIRAMINE POLISTIREX, GINKGO, CRX-119, AMINEPTINE, DEBRISOQUIN, BICIFADINE, MMDA, MIANSERIN, TAPENTADOL, TRAMADOL, BUPROPION, LEVOMILNACIPRAN, ATOMOXETINE, CITALOPRAM, CLOMIPRAMINE, DESIPRAMINE, DESVENLAFAXINE, DEXTROAMPHETAMINE, DOTHIEPIN, DULOXETINE, IMIPRAMINE, LOFEPRAMINE, METHYLPHENIDATE, MAPROTILINE, MILNACIPRAN, DIETHYLPROPION, NEFAZODONE, NISOXETINE, AMITRIPTYLINE, NORTRIPTYLINE, VENLAFAXINE, PHENELZINE, PROTRIPTYLINE, QUETIAPINE, PSEUDOEPHEDRINE, PHENTERMINEGUANADREL SULFATE, ZOTEPINE, AMITIFADINE, EDIVOXETINE, BETHANIDINE, TRAZODONE, DEXMETHYLPHENIDATE, PETHIDINE, PAROXETINE, TEDATIOXETINE, TESOFENSINE, PHENTERMINE, PROTRIPTYLINE MIRTAZAPINE, DEXTROMETHORPHAN, METHAMPHETAMINE, NORTRIPTYLINE, AMOXAPINE, TRIMIPRAMINE, DOPAMINE, SIBUTRAMINE, CHLORPHENAMINE, ORPHENADRINE, ESCITALOPRAM, KETAMINE, EPHEDRA, EPHEDRINE, GINKGO BILOBA, XEN2174, 3,4-METHYLENEDIOXYMETHAMPHETAMINE, PHENDIMETRAZINE, LEVONORDEFRIN, REBOXETINE GLYCINE, BITOPERTIN, CHEMBL88588, CILNIDIPINE
8	*IGSF11*	3.71	
9	*CACNA1B*	3.71	VERAPAMIL, GABAPENTIN, LEVETIRACETAM, Z160, AGMATINE, CELECOXIB, CNV2197944, VERAPAMIL, GABAPENTIN ENACARBIL, AMLODIPINE, AGMATINE, SAFINAMIDE, RALFINAMIDE, ZICONOTIDE, BEPRIDIL HYDROCHLORIDE, CLEVIDIPINE, ELPETRIGINE, ATAGABALIN, ZICONOTIDE ACETATE, IMAGABALIN, PREGABALIN, AMLODIPINE, GABAPENTIN, LEVETIRACETAM, CILNIDIPINE, NITRENDIPINE
10	*DLK2*	3.49	

Note: Where applicable, each gene is accompanied by a list of drugs that were associated with it in at least one of the following databases: DrugBank, PharmGKB, ClinicalTrials.gov, DGIdb, and FDA Approved Drugs. Standard deviation of the expression values at the Log2FC level was <0.002.

### Gene-Gene Interaction Network

To conduct the gene-gene (G-G) functional interaction analysis of key biomarkers, we used the GeneMANIA online software. All membrane protein-encoding genes (Supplementary Table S2) were used as input data for G-G network. To find the hub genes, we ranked genes by the number of edges they shared with other genes and the difference in expression levels between SCC and AC.

**FIGURE 4:**
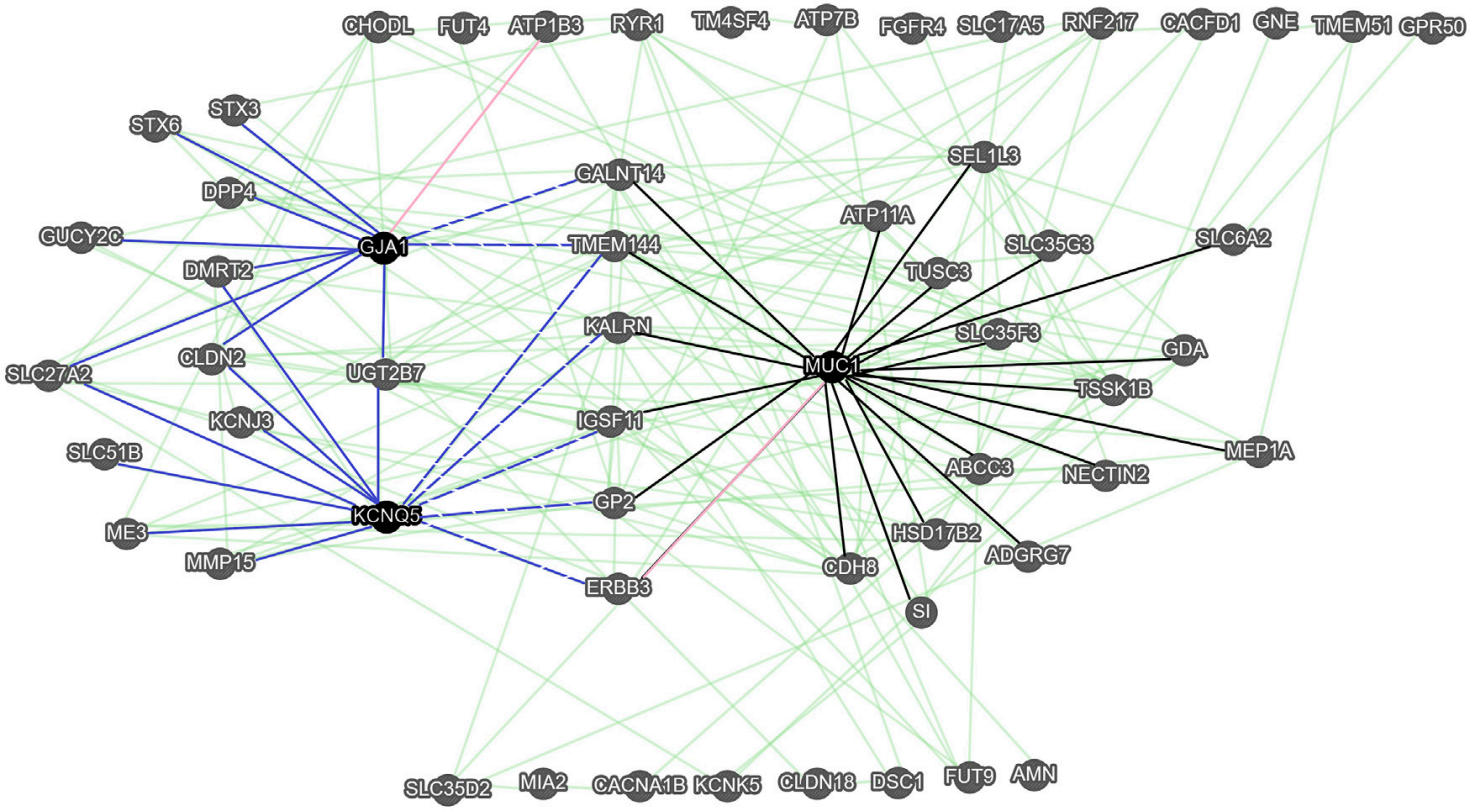
The gene-gene interaction network of membrane protein-encoding genes obtained with GeneMANIA. Green edges correspond to the functional associations between genes (nodes), while pink edges represent the predicted gene-gene interaction. The black edges correspond to genes functionally associated with the MUC1 hub gene (sub-network 1). The solid blue edges correspond to genes functionally associated with the KCNQ5 and GJA1 hub genes (sub-network 2), while the blue dashed edges correspond to genes functionally associated with the KCNQ5, GJA1, and MUC1.

The functional associations between 57 of 84 genes were observed (Figure 4). Three genes, namely the mucin 1 (*MUC1*), the gap junction protein alpha 1 (*GJA1*), and *KCNQ5* with a high number of gene-gene interactions (more than 11 edges) and a high difference in expression levels between SCC and AC (abs (Log2FC) > 2.8) were considered as hub genes. Within the 57-gene network, we identified two sub-networks. In the first sub-network, the *MUC1* gene is a primary hub gene linked with 21 significant genes, such as *GALNT14, ABCC3,* and *ERBB3*. In the second sub-network, there are *KCNQ1* and *GJA1* hub genes linked with 12 significant genes, such as *GUCY2C*, and ERBB3. Seven genes are shared by both sub-networks (*GALNT14, TMEM144, KALRN, IGSF11, GP2*, and *ERBB3*).

## Discussion

In this study, we aimed to identify the most informative molecular markers to distinguish between SCC and AC and to characterize pertinent genes in terms of their potential utility in individualized cancer treatment. We used a robust two-step protocol for identifying the most informative RNA-seq biomarkers important for cancer diagnosis and potentially druggable genes crucial for SCA treatment.

### Informative Biomarkers

SCC and AC significantly differ at the molecular level (Lin et al., 2017; Network, 2017). Previous studies have confirmed these results, and owing to the use of a more sensitive approach based on ensemble FS, even stronger differences have become known. We herein identified 8246 DEGs, of which, 5434 (65.9%) have not been mentioned in previous studies (Greenawalt et al., 2007; Lin et al., 2017). Further, 81.7% of the 3443 genes identified by Lin *et al.* (Lin et al., 2017) and 64.7% of the 546 genes identified by Greenawalt *et al.* (Greenawalt et al., 2007) were identified in this study. These differences could be attributed to (1) the absence of some genes in our dataset and (2) using a more stringent method for multiple testing correction (for Ttest, MDFS-1D, and MDFS-2D).

We observed that for different FS methods, the overlap between the sets of selected biomarkers was low (Supplementary Figures S1, S5). This is a manifestation of the well-known problem that different selection methods tend to produce different biological signatures (Abusamra, 2013). Such differences can also be due to different approaches for FS implemented in different algorithms (Bommert et al., 2020). Furthermore, application of methods that reduce redundancy in the feature set result in decreased stability of the set of biomarkers (Polewko-Klim and Rudnicki, 2020). Nevertheless, as per ontological analyses, biological functions captured by different gene subsets are rather similar (Dessi et al., 2013). In this study, we constructed highly effective predictive models (AUC >0.994) using only top 20 features returned by any of the applied FS methods. Overlooking genes that are important for biological and functional interpretation of differences between datasets is possible when only one FS method is used for identifying relevant features. Thus, the application of diverse FS methods is highly recommended.

Among the 294 genes that facilitated differentiation between AC and SCC, 87 have not been previously reported as relevant (Greenawalt et al., 2007; Lin et al., 2017). These newly discovered relevant genes can potentially be used as drug targets.

Further, although subtypes can be easily determined by pathologists, these genes can facilitate automatic pathological identification in clinical settings with extremely high sensitivity and specificity. The set of genes presented in Supplementary Table S1 can be used for SCC and AC diagnosis.

### Specific Therapeutic Agents for SCC and AC

We focused on membrane protein-encoding genes that were drug targets for chemotherapy and immunotherapy and involved in pharmacokinetics and pharmacodynamics pathways. *ERBB3, ATP7B, ABCC3,* and *GALNT14* were particularly interesting, as genes encoding them are already related to 11 common anti-SCA drugs (Figure 3). Both, overexpression (*GALNT14*) or under-expression (*ERBB3, ATP7B, ABCC3*) of these genes in SCC relative to AC may contribute to a different response to these common anticancer drugs. Interestingly, according to GeneCards, *GALNT14* is overexpressed by > 5-fold in the esophagus–mucosa in normal tissues.

Considering the upregulated expression of *GALNT14* in SCC *vs*. AC, *GALNT14* appears to be a promising potential therapeutic target for SCC. *GALNT14* is an antitumor agent and therapeutic response predictor for concurrent chemoradiotherapy wherein the platinum-based drugs fluorouracil and cisplatin are used for advanced SCC (Honing et al., 2014; Tsou et al., 2017). Gebski et al. (2007) reported that neoadjuvant chemotherapy with cisplatin and 5-fluorouracil led to relatively better survival of patients with AC. *GALNT14* genotype is also a potential predictor of the response to the first course of 5-fluorouracil, mitoxantrone, and cisplatin chemotherapy in patients with advanced hepatocellular carcinoma (Liang et al., 2011). The overexpression of *GALNT14* is a strong biomarker correlated to the sensitivity of Apo2L/TRAIL-based anticancer therapy. *GALNT14* alters cell migration and cellular morphology, and its overexpression causes malignancies, such as those of the breast, ovarium, lungs, and skin (Erdal et al., 2017), so it is a good predictor of therapeutic outcomes, particularly of chemotherapy, in multiple cancers (Lin and Yeh, 2020).

*ERBB3, ATP7B*, and *ABCC3* are also reportedly promising drug targets. *ERBB3 (HER3)* is a member of the epidermal growth factor receptor family of receptor tyrosine kinases. A comprehensive analysis of *EGFR, HER2,* and *HER3* coexpression and dimerization that were observed in the two histopathological subtypes of SCA has been previously performed (Fichter et al., 2014). Fichter *et al.* (Fichter et al., 2014) suggested that preclinical investigations of antibody-dependent cellular cytotoxicity elicited by trastuzumab and pertuzumab can be very important in AC, namely, these drugs indicate an effect in AC cancer cells with high *HER2* expression and *HER2* homodimers. *ATP7B* is a key mediator of cellular cisplatin, carboplatin, and oxaliplatin accumulation, these platinum-based drugs are widely used in modern cancer therapeutics (Li et al., 2016). Li *et al.* reported that *ATP7B* overexpression plays a key role in platinum resistance in SCC (Li et al., 2016). *ABCC3* is a transporter and inducer of cisplatin and an inhibitor of doxorubicin. It is involved in cellular resistance to chemotherapy with fluorouracil in patients with SCC (Zhou et al., 2008) and is also a putative biomarker of resistance to antimitotic agents, such as paclitaxel, used in breast cancer treatment (O'Brien et al., 2008).

### Novel Drug-Related Genes

We also detected several new drug-related genes with a high Log2FC value, which were associated with cancer promotion, transformation, and progression (Supplementary Table S2) and thus relevant for targeted treatment of SCA. Three genes were under-expressed in SCC *vs*. AC, namely claudin 18 (*CLDN18*), guanylyl cyclase C (*GUCY2C*), and fibroblast growth factor receptor 4 (*FGFR4*), and two genes were overexpressed, namely potassium voltage-gated channel subfamily Q member 5 (*KCNQ5*) and calcium voltage-gated channel subunit alpha1 B (*CACNA1B*). These genes showed a high difference in their expression levels between SCC and AC and are already associated with certain drugs. For example, the tight junction molecule claudin-18 isoform 2 (*CLDN18.2*) is a target for claudiximab, which is a first-in-class chimeric monoclonal antibody used for the treatment of gastric cancer (Singh et al., 2017). Aka *et al.* (Aka et al., 2017) reported that *GUCY2C* is a potentially ideal target antigen for colorectal cancer immunotherapy and that supplementation with linaclotide (*GUCY2C* ligand) is a novel and promising strategy for tumor prevention. Zhongwei et al. (Xin et al., 2018) suggested that blocking *FGFR4* significantly suppressed the malignant behaviors of SCC, indicating that *FGFR4* is a potential target for SCC treatment. *KCNQ5* interacts with celecoxib and is a promising drug for prevention/treatment of several cancers, such as colon, breast, prostate, and head and neck cancers (Toloczko-Iwaniuk et al., 2019). *CACNA1B* is useful for evaluating the efficacy of chemoradiotherapy against SCC (Sasaki et al., 2017).

Considering the significant differences in gene expression levels of *ERBB3, ATP7B, ABCC3, GALNT14, CLDN18, GUCY2C, FGFR4, KCNQ5,* and *CACNA1B* between patients with SCC and AC, we recommend conducting further preclinical research on them. Future studies are warranted to investigate how these genes can be used to develop more effective chemotherapy and immunotherapy treatment methods for patients with SCA, as well as options for novel drug use associated with those genes with large fold change in SCC and AC.

### Gene-Gene Interaction

The G-G interaction analysis via GeneMANIA indicated three pivotal hub genes, namely *MUC1, GJA1*, and *KCNQ5*. The most linked of them, the *MUC1*, is an oncogene that exhibits extensive glycosylation *in vivo*. The aberrant glycosylation and overexpression of *MUC1* gene in cancer cells may lead to cancer invasion, metastasis, angiogenesis, and apoptosis by virtue of its participation in intracellular signaling processes and the regulation of related biomolecules (Chen et al., 2021). Moreover, mucin 1 protein coded by *MUC1* is an important barrier to the penetration of drugs and takes part in the inhibition of apoptosis in tumor cells. It has been widely recognized as one of the most promising molecular targets in cancer therapy (Lee et al., 2021). For example, the overexpression of this membrane-bound glycoprotein limits the effectiveness of 5-fluorouracil treatment in patients with pancreatic cancer (Kalra and Campbell 2009), and decreases sensitivity of cisplatin in SCC (Zhao et al., 2021). Mucin 1 plays a key role in trastuzumab resistance in breast cancer (Hosseinzadeh et al., 2022). The high-expression of *MUC1* is associated with a poor prognosis for esophageal cancer patients (Song et al., 2003), contributes to SCC metastasis (Ye et al., 2011), and plays a pivotal role in the progression to AC (Adil Butt et al., 2017).

*GJA1* and *KCNQ5* are hub genes of one gene module and both are significantly up-regulated in SCC *vs*. AC. *GJA1* (encoding *Cx43*) is a member of the connexin family that possesses both tumor-suppressive, and oncogenic functions (Aasen et al., 2019). The misregulation of connexins affects a process of cell differentiation, inflammation, and cell death (Katturajan and Evan Prince, 2021). *GJA1* is a highly attractive target for delivering drugs directly into the cytoplasm of cancer cells, due to the permeability of gap junction channels to small molecules and macromolecules (Bonacquisti and Nguyen, 2019). The silencing of *GJA1* gene may cause a reduction of paclitaxel efficiency in gastric cancer (Zhao et al., 2019), and cisplatin-resistance in lung cancer (Luo et al., 2021). The high *GJA1* expression in SCC cancer cells is associated with poor survival of patients (Tanaka et al., 2016). The exact role of *KCNQ5* in SCA cancer tumor genesis and progression is not known. But recent studies have shown that this oncogene is a potential prognostic biomarker for gastrointestinal cancer (Shorthouse et al., 2020) and a promising biomarker for early colorectal cancer detection (Cao et al., 2021). Considering the over-expression of *GJA1* and *KCNQ5* in SCC *vs*. AC, they can be promising molecular targets for SCC.

## Data Availability

Data from TCGA is publicly available. The datasets presented in this study can be found in an online repository, http://www.biosino.org. The data accession number is OEP000138. NODE-SCC data is available upon reasonable request from the corresponding author through the repository.
