# Supplementary figures and images for: Identification of Candidate Therapeutic Genes for More Precise Treatment of Esophageal Squamous Cell Carcinoma and Adenocarcinoma
Source: Front Genet. 2022 May 19;13:844542. doi: 10.3389/fgene.2022.844542 (PMC9161154; doi:10.3389/fgene.2022.844542)

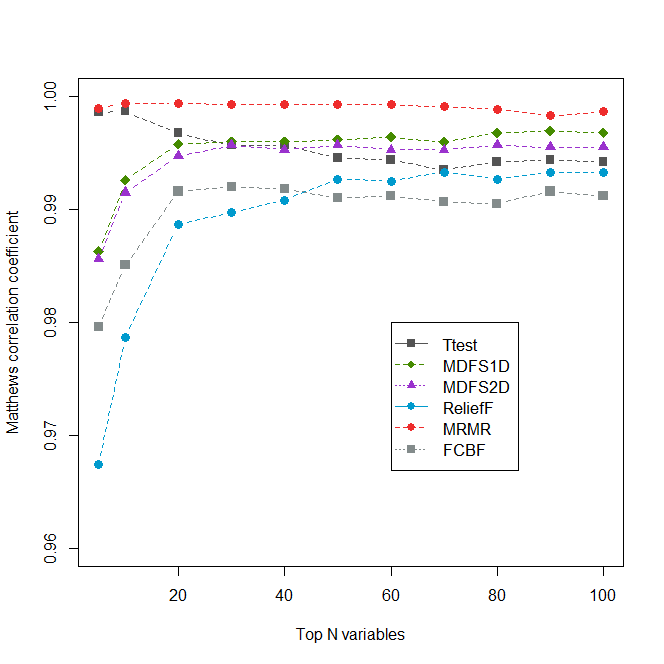

Supplement: Supplementary file 1 [file Image3.TIF]

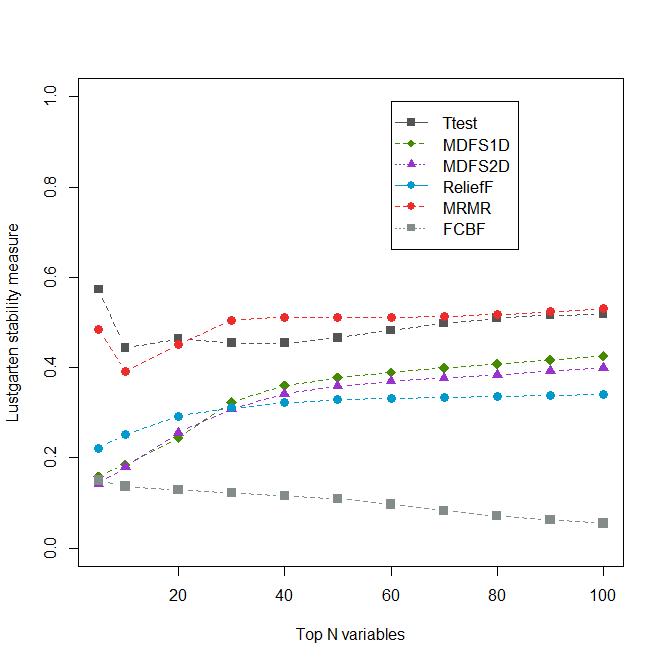

Supplement: Supplementary file 2 [file Image4.TIF]

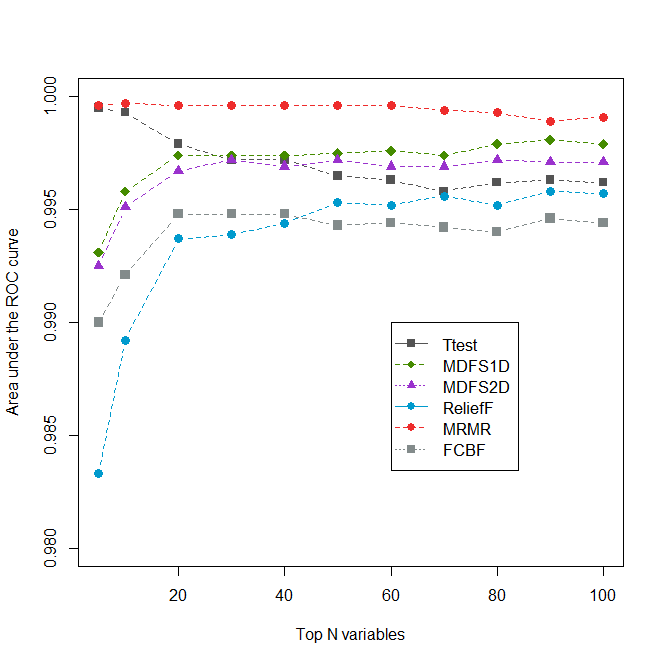

Supplement: Supplementary file 3 [file Image2.TIF]

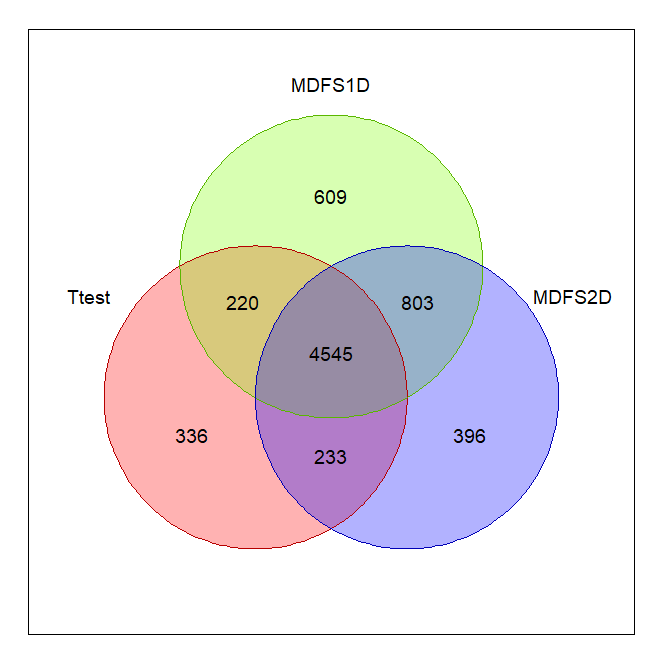

Supplement: Supplementary file 4 [file Image1.TIF]

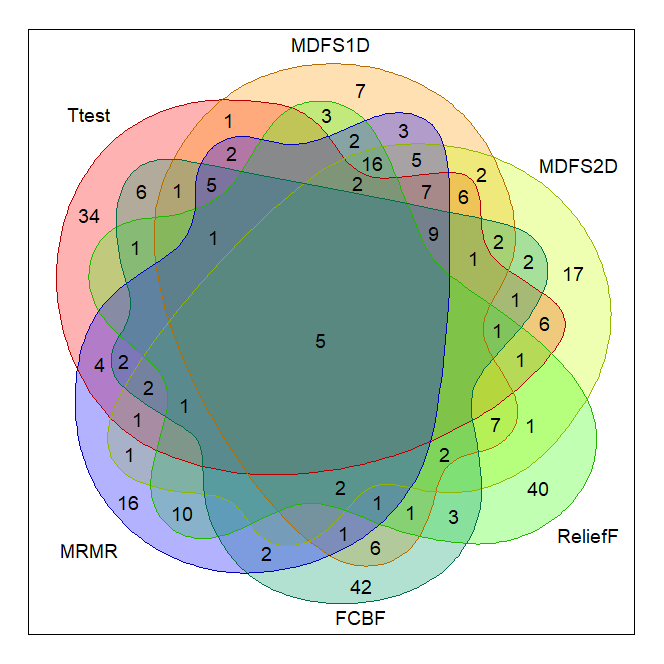

Supplement: Supplementary file 6 [file Image5.TIF]
