# Supplementary material for: Identification of Candidate Therapeutic Genes for More Precise Treatment of Esophageal Squamous Cell Carcinoma and Adenocarcinoma
Source: Front Genet. 2022 May 19;13:844542. doi: 10.3389/fgene.2022.844542 (PMC9161154; doi:10.3389/fgene.2022.844542)
Supplement: Supplementary file 5 [file Table1.DOC]

**Supplementary Material**

**1 NODE-SCC sample preparation and data collection procedure**

For RNA-seq, total RNA (>18 nucleotides) was extracted from tissues using the miRNeasy Mini Kit with DNase treatment (Qiagen) according to the manufacturer’s protocol. RNA quality was evaluated using the Agilent 2100 Bioanalyzer (Agilent Technologies). All RNA samples had RNA integrity numbers (RINs) greater than 7.5. Libraries for both tumor and normal tissues were prepared using [Ribo-Zero rRNA Removal Kit](https://support.illumina.com/content/dam/illumina-support/documents/documentation/chemistry_documentation/ribosomal-depletion/ribo-zero/ribo-zero-reference-guide-15066012-02.pdf) coupled with the Illumina RNA-seq library protocol. The quality of the cDNA libraries was checked using an Agilent Bioanalyzer and DNA 1000 kit. All libraries were quantified with a Qubit 2.0 Fluorometer (Life Technologies) and stored in non-stick Eppendorf tubes (Life Technologies) at −20°C. One of the tumor RNA libraries (ESCC_15_54_T) failed to pass our quality control due to RNA degradation (concentration 3.9 ng/µl, A260/230 value 0.07). The remaining 89 RNA samples were sequenced using the Illumina HiSeq 2500 platform.

The raw sequencing reads were processed with Trimmomatic (v0.35) (Bolger et al., 2014) to remove sequencing adapter and low-quality data. The high-quality RNA-seq reads were mapped to the human reference genome (GRCh37, version 19) using HiSat2 (v2.0.5) (Kim et al., 2015) with default parameters. The featureCounts algorithm (Liao et al., 2014) were used for the quantification of RefSeq genes (hg19). The raw counts of each gene were normalized to the reads per million sequenced reads then transformed to the log2 scale. The differentially expressed genes were identified by comparing gene expression in tumor versus normal samples. We used fold change ≥ 1.50 or ≤ 0.67 and adjusted (Benjamini-Hochberg method (Benjamini and Hochberg, 1995)) p < 0.05 as criteria to select the differentially expressed coding genes.

**2 Feature selection methods**

Nowadays the number of available biomarker selection methods is growing (Ang et al., 2016) and it is an important procedure especially in biomarker discovery from high-dimensional genomics data. Using a single feature selection method gives the high classification accuracy, however this approach may lead to missing out important biological information. Therefore, in current study, identification of the most informative variables from the COM-SCA data was realized by using the ensemble feature selection method based on six different FS methods: Ttest, MDFS1D, MDFS2D, FCBF, ReliefF, and MRMR.

In the first method, the Ttest with unequal population variances is performed, where the two populations correspond to the two esophageal cancer subtypes, SCC as positive class and AC as negative class. Multiple testing corrections is performed by using Hochberg procedure (Hochberg and Benjamini, 1990), one of the common procedures in genomics analysis (Goeman and Solari, 2014). In the second method, reduction and ranking of biomarkers is performed by using the multi-dimensional feature selection (MDFS) which is based on mutual information between a decision variable (the esophageal cancer subtypes) and single descriptive variable (MDFS1D), or pairs of descriptive variables (MDFS2D) that allows to identify the variables that gain importance due to interactions with other variables. The analysis is performed by using “MDFS” R package (Piliszek et al., 2019). Similarly, the Hochberg multiple testing corrections is used to counteract the problem of multiple comparisons. In the third method, the FCBF method based on correlation as efficient way of analyzing feature redundancy is used. It is implemented in “Biocomb” R package (Novoselova N et al., 2018). The other two used feature selection methods are ReliefF implemented in “CORElearn” R package (Robnik-Sikonja M and P, 2018) and MRMR implemented in “mRMRe” R package (De Jay N, 2017). Both methods only rank variables but do not filter out. ReliefF is a feature weighting algorithm that is sensitive to feature interactions (Kira Kenji and Larry., 1992). Two different types of distance between nearest neighbor instance pairs (ReliefFexpRank and ReliefFbestK) are compared. The MRMR method is based on mutual information as a measure of the relevancy and redundancy, where the redundancy of a selected feature subset is an aggregate mutual information measure between each pair of features in the selected feature and the relevancy to a class is an aggregate mutual information measure between each feature with respect to the class (Ang et al., 2016).

3. Classification algorithm
